# Impact of vaccination and non-pharmacological interventions on COVID-19: a review of simulation modeling studies in Asia

**DOI:** 10.3389/fpubh.2023.1252719

**Published:** 2023-09-25

**Authors:** Karan Thakkar, Julia Regazzini Spinardi, Jingyan Yang, Moe H. Kyaw, Egemen Ozbilgili, Carlos Fernando Mendoza, Helen May Lin Oh

**Affiliations:** ^1^Vaccine Medical Affairs, Emerging Markets, Pfizer Inc., Singapore, Singapore; ^2^Vaccine Medical Affairs-Emerging Markets, Pfizer Inc., São Paulo, Brazil; ^3^Vaccine Global Value and Access, Pfizer Inc., New York, NY, United States; ^4^Vaccine Medical Affairs, Emerging Markets, Pfizer Inc., Reston, VA, United States; ^5^Asia Cluster Medical Affairs, Emerging Markets, Pfizer Inc., Singapore, Singapore; ^6^mRNA Vaccine Value & Evidence, Emerging Markets, Pfizer Inc., Mexico City, Mexico; ^7^Department of Infectious Diseases, Changi General Hospital, Singapore, Singapore

**Keywords:** COVID-19, vaccination, epidemiological modeling, Asia, intervention

## Abstract

**Introduction:**

Epidemiological modeling is widely used to offer insights into the COVID-19 pandemic situation in Asia. We reviewed published computational (mathematical/simulation) models conducted in Asia that assessed impacts of pharmacological and non-pharmacological interventions against COVID-19 and their implications for vaccination strategy.

**Methods:**

A search of the PubMed database for peer-reviewed, published, and accessible articles in English was performed up to November 2022 to capture studies in Asian populations based on computational modeling of outcomes in the COVID-19 pandemic. Extracted data included model type (mechanistic compartmental/agent-based, statistical, both), intervention type (pharmacological, non-pharmacological), and procedures for parameterizing age. Findings are summarized with descriptive statistics and discussed in terms of the evolving COVID-19 situation.

**Results:**

The literature search identified 378 results, of which 59 met criteria for data extraction. China, Japan, and South Korea accounted for approximately half of studies, with fewer from South and South-East Asia. Mechanistic models were most common, either compartmental (61.0%), agent-based (1.7%), or combination (18.6%) models. Statistical modeling was applied less frequently (11.9%). Pharmacological interventions were examined in 59.3% of studies, and most considered vaccination, except one study of an antiviral treatment. Non-pharmacological interventions were also considered in 84.7% of studies. Infection, hospitalization, and mortality were outcomes in 91.5%, 30.5%, and 30.5% of studies, respectively. Approximately a third of studies accounted for age, including 10 that also examined mortality. Four of these studies emphasized benefits in terms of mortality from prioritizing older adults for vaccination under conditions of a limited supply; however, one study noted potential benefits to infection rates from early vaccination of younger adults. Few studies (5.1%) considered the impact of vaccination among children.

**Conclusion:**

Early in the COVID-19 pandemic, non-pharmacological interventions helped to mitigate the health burden of COVID-19; however, modeling indicates that high population coverage of effective vaccines will complement and reduce reliance on such interventions. Thus, increasing and maintaining immunity levels in populations through regular booster shots, particularly among at-risk and vulnerable groups, including older adults, might help to protect public health. Future modeling efforts should consider new vaccines and alternative therapies alongside an evolving virus in populations with varied vaccination histories.

## 1. Introduction

The SARS-CoV-2, which causes COVID-19, rapidly spread from the first reported symptoms in Wuhan, China, in December 2019 through to the declaration of an International Public Health Emergency by the World Health Organization in January 2020 (1, 2). The nature of the virus has since changed through its evolution into new variants of concern, with differing potentials for infection, severe disease course, and negation of immunity from vaccination or prior infection (3, 4). The human response to the pandemic has also developed from the implementation of non-pharmacological countermeasures through to pharmacological and vaccination strategies, which have mitigated the impact of the pandemic with varying degrees of effectiveness (5, 6).

In the early pandemic, non-pharmacological interventions were implemented across many regions to ease the immediate disease burden of COVID-19 and prevent the overwhelming of public health systems. Although lockdowns and school/workplace closures were effective in reducing transmission and patient healthcare costs, the economic disruption, declining mental health, and social discontent caused by these interventions raised concerns (7–9). The development and wider rollout of effective vaccines has further changed the dynamics of the situation (10–13). In the context of limited vaccine supply, the WHO recommended administration strategies that aimed to reduce mortality from COVID-19 infection (14). Individuals at risk of infection and/or severe disease were targeted, namely, front-line healthcare workers and older adults, the latter group being at particular risk of morbidity and mortality from COVID-19, compared with younger age groups (6, 15, 16). As supplies have stabilized, campaigns to vaccinate the wider public have faced challenges in the form of hesitancy among certain populations to receive initial doses of the vaccine and/or subsequent boosters (17, 18). Additionally, from 2022, the progression from a prevalent Delta to Omicron variant has shifted the burden of disease to younger people, including pediatric patients in Asia (19–21).

Ongoing high infection rates, an evolving virus, vaccine fatigue, and the relaxation of pandemic countermeasures suggest that COVID-19 will pose ongoing challenges to public health with potential differential effects across age groups (22, 23). Owing to large and diverse populations with different cultural practices and healthcare systems, the development of the COVID-19 situation in Asian settings is likely to pose unique questions for healthcare professionals and policymakers in the region. The dynamics of vaccination against SARS-CoV-2 are complicated by age-dependent factors, type of vaccines used and population vaccine coverage, changing levels of infection and the prevailing strains of concern, and the relaxation of non-pharmaceutical interventions, hence, necessitating the use of mathematical modeling studies in Asia.

Throughout the crisis, epidemiological models have been widely developed and used by policymakers to estimate the impact of interventions on projected disease burden and demands on public healthcare systems (24, 25). Mitigation strategies, including the mandating of mask-wearing, social distancing, restrictions on movement and gatherings, through to pharmaceutical and vaccination strategies, have been implemented to control the spread of virus with varying degrees of effectiveness (5, 6). Although models cannot exactly predict key factors such as the basic reproduction number (R_0_), these tools have been applied both globally and in Asia to guide potential care needs in terms of stratifying risk, directing limited resources, and planning for future outbreaks (26, 27). Models of increasing complexity have also been developed to account for the dynamics of transmission among different age groups in the context of different vaccination statuses and waning vaccine effectiveness over time or against emerging variants (28).

Mechanistic models are formulated to mimic the nature of spreading diseases, allowing the simulation of the complex dynamics and nonlinear feedback of COVID-19 transmission within a population (29). An example is the Susceptible-Infected-Exposed-Removed (SEIR) compartmental model, which considers interactions among cohorts of a population, categorized according to disease status. Individuals within a compartment are not differentiated from one another but can flow from one compartment to another at rates defined by parameter inputs. This approach can be effective for examining disease dynamics at a macro level. As a type of mechanistic model, individual-level models treat members of a population as unique agents; this may better capture phenomena such as super-spreader events. Statistically derived models (for example, distribution fitting and regression-type analysis) can also be applied to accurately characterize data from a small number of parameters and are suitable for short-term forecasts that are accurate, repeatable, and sensitive to momentary changes in a system (30). These tools include regression using least-squares or Bayesian estimations. These features make statistical models valuable for producing up-to-date estimates of COVID-19’s underlying nature as a disease (e.g., the basic reproduction rate) and the demographic patterns of the population (e.g., how long people self-isolate). Although statistical models can be highly accurate, they treat the situation as a black box and do not mimic the underlying nature of the disease or its consequences. Thus, these models are not well suited for long-term projections or for exploring hypothetical scenarios.

This review aims to examine published COVID-19 epidemiological modeling studies conducted in Asian settings and assess the models used and their findings regarding the impact of vaccination and non-pharmacological measures on COVID-19 infections, hospitalization, mortality, and policies. We discuss the implications of modeling studies in Asia in the context of the latest data on COVID-19 vaccines, the emergence of SARS-CoV-2 variants, and regional vaccination policies.

## 2. Methods

### 2.1. Literature search

For the purposes of this study, eligible reports examined the infection, mortality, hospitalization, vaccination, and/or policy outcomes from computational models for COVID-19 set in Asia. We followed the PRISMA guidelines in conducting systematic elements of this review. A literature search in the PubMed database for peer-reviewed, published, and accessible articles in English, available from the start of the COVID-19 pandemic, was conducted on 9 November 2022. Search terms focused on keywords identified from PICO (populations, intervention, comparison, outcome) elements and included terms common among modeling studies (e.g., Susceptible-Infected-Recovered, simulation, agent-based), types of outcomes (e.g., hospitalization, infection, and mortality) and the target interventions (e.g., vaccination) (See Supplementary Table S1 for full search terms). The search was filtered for articles that had available full text, and the results were exported to Microsoft Excel for initial screening based on the titles and abstracts. Articles that did not include an epidemiological model (e.g., laboratory and clinical studies) and/or were not concerned with COVID-19 (e.g., other diseases examined within a COVID-19 setting) were excluded.

Screened articles were further categorized based on the abstract and display items to identify a smaller set of articles that fulfilled any of the following conditions: (i) the article mentions collaborations with, or recommendations to, policy-makers; or (ii) the article considers the effects size of pharmacological and/or non-pharmacological interventions on any of the outcomes of interest (infection, mortality, hospitalization, and/or vaccination).

### 2.2. Data extraction

One research assistant performed data extraction for model classification, location, time, and scale, interventions applied, consideration of age groups, and impacts of interventions on rates of infection, hospitalization/severe disease, mortality, and/or policy. Another research assistant confirmed the extracted data.

Non-pharmacological interventions were categorized as “reducing contact” (measures that reduce transmission from infected individuals, such as lockdowns, school/workplace closure, and social distancing), “border control” (preventing infected individuals from entering the population; for instance, airport quarantine and border closure), “contact tracing” (testing/quarantining of people having close contact with infected individuals), “hygiene” (e.g., hand washing, ventilation), “PPE” (e.g., wearing of masks, gloves), and “other.” In categorizing models employed in reports, we differentiated between “mechanistic” (i.e., any model that applied *a priori* mathematical equations describing physical systems, with or without stochastic elements) and “statistical models” (e.g., models based on regression or Bayesian analysis, or machine learning of data sets), and classified combinations as “both.” We further categorized mechanistic models as compartmental (cohort), agent-based (individual) or combination models (combining elements from both types).

## 3. Results

### 3.1. Study settings

**Figure 1:**
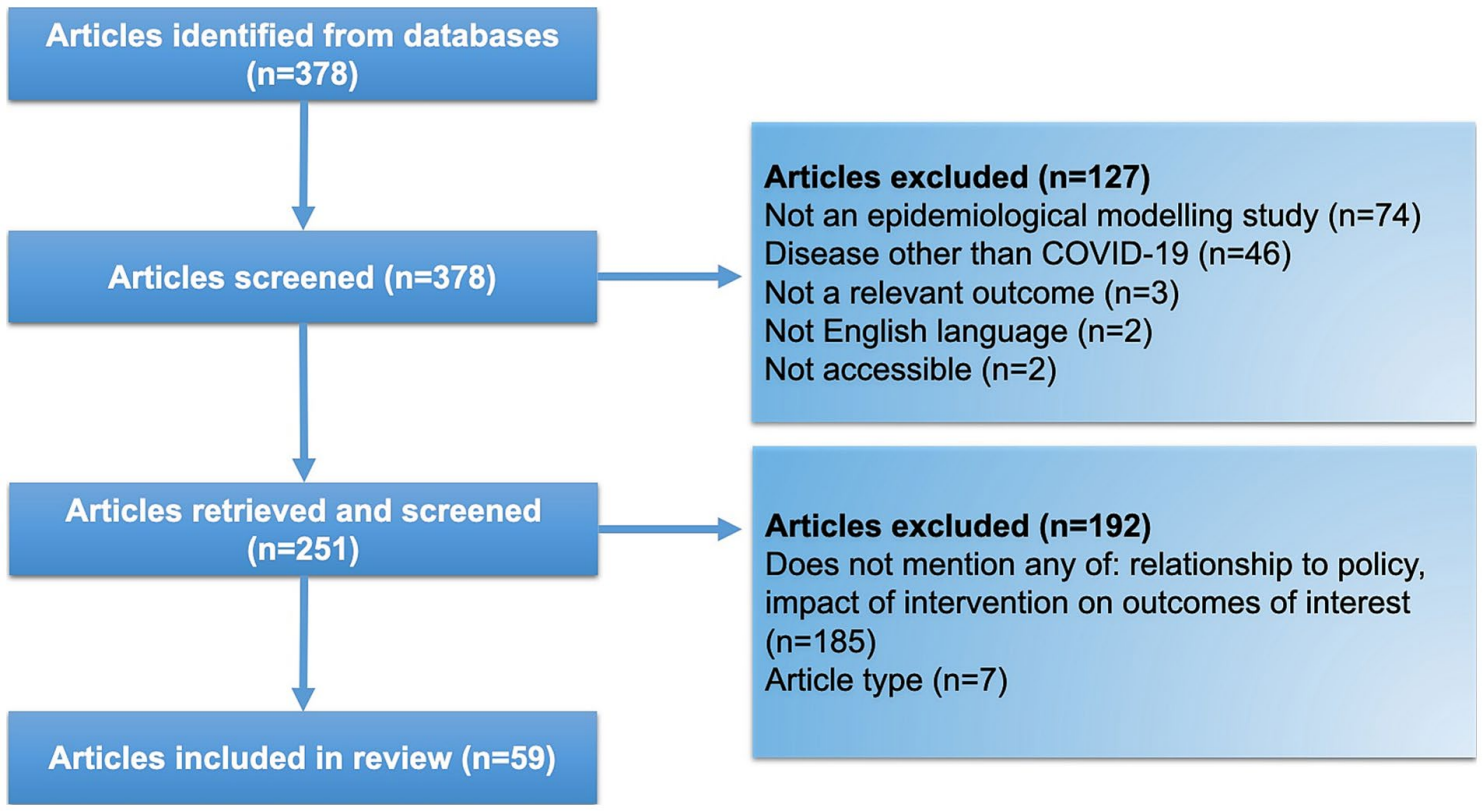
Literature search flow diagram.

The literature search returned 378 results, which, after exclusions for irrelevance (*n* = 127) and ineligibility (*n* = 192), resulted in a set of 59 reports selected for data extraction (Figure 1; Table 1). Most reports were from East Asia (China, 21/59 [3 of which were set in Hong Kong], 35.6%; Japan, 7/59, 11.9%; South Korea 6/59, 10.2%), followed by South Asia (India, 5/59, 8.5%; Bangladesh, 3/59, 5.1%; Sri Lanka, 1/59, 1.7%) and South-East Asia (Malaysia, 3/59, 5.1%; the Philippines, 2/59, 3.4%; Thailand, 1/59, 1.7%; Vietnam; 1/59, 1.7%). Multiple countries were considered in 6.8% (4/59) of reports, and 5.1% (3/59) presented modeling results that examined specific Asian regions as part of a global setting. The majority of reports (59.3%, 35/59) applied a modeling start date in 2020, with fewer studies starting in 2021 (33.9%, 20/59) and 2022 (3.4%, 2/59).

**Table 1:** Key characteristics of included reports (*N* = 59 studies).

Author Year (citation)	Locality	Intervention			Model type		Age groups	Outcomes		
		Nonpharmacological	Pharmacological	Vaccination efficacy parameters^a^				Infection	Hospitalization and severe disease	Mortality
Baniasad 2021 (31)	Iran, Turkey, Saudi Arabia, United Arab Emirates, India, Russia, Philippines, South Korea	Contact tracing, reducing contact	Vaccination (unspecified)	Unspecified	Both	Combination	N/A	X	X	X
Cai 2022 (32)	China	Reducing contact	Vaccination (Sinovac/CoronaVac)	51.8%, as a variable	Mechanistic	Compartmental	14 age groups	X	X	X
Ferguson 2022 (33)	Bangladesh	Reducing contact, PPE	N/A	N/A	Mechanistic	Compartmental	N/A	X	X	X
Jung 2020 (34)	North Korea	Reducing contact	Vaccination (unspecified)	50–80% in reducing susceptibility 80–95% reducing hospitalization	Mechanistic	Compartmental	N/A	X	X	
Leung 2021 (35)	Global, Hong Kong, China	Border control	Vaccination (unspecified)	Weighted average of available vaccines	Mechanistic	Compartmental	Age as variable	X	X	X
Qian 2022 (36)	China	Contact tracing, reducing contact	Vaccination (unspecified)	Unspecified	Mechanistic	Compartmental	N/A	X	X	X
Suphanchaimat 2021 (37)	Thailand	N/A	Vaccination (unspecified)	50% against infection	Mechanistic	Compartmental	N/A	X	X	X
Cai 2022 (38)	China	N/A	Vaccination (unspecified)	Variable	Mechanistic	Compartmental	16 age groups	X	X	
Ediriweera 2020 (39)	Sri Lanka	Reducing contact	N/A	Weighted average of available vaccines	Mechanistic	Compartmental	N/A	X	X	
Kong 2022 (40)	Japan, China	Contact tracing, border control, reducing contact	Vaccination (unspecified)	Unspecified	Mechanistic	Compartmental	N/A	X	X	
Rajput 2021 (41)	India	Contact tracing, reducing contact	Vaccination (unspecified)	80%, variable	Mechanistic	Compartmental	N/A	X	X	
Shah 2022 (42)	India	Reducing contact	N/A	N/A	Mechanistic	Combination	N/A	X	X	
Shankaranarayanan 2022 (43)	India	Reducing contact	Vaccination (unspecified)		Statistical	N/A	N/A	X	X	
De Lara-Tuprio 2022 (44)	The Philippines	Contact tracing, reducing contact, hygiene, PPE	N/A	N/A	Mechanistic	Compartmental	N/A	X		
Dong 2022 (45)	China	Reducing contact	N/A	N/A	Mechanistic	Agent-based	Age as variable	X		
Foy 2021 (46)	India	Reducing contact	Vaccination (unspecified)	0–100%	Mechanistic	Compartmental	7 age groups	X		X
Fu 2022 (47)	China	N/A	Vaccination (CoronaVac/BBIBP-CorV)	Unspecified	Statistical	N/A	3 age groups	X	X	
Gaudou 2020 (48)	Vietnam	Reducing contact, PPE	N/A	N/A	Mechanistic	Combination	Age as variable	X		X
Ko 2021 (49)	South Korea	Border control, reducing contact	Vaccination (unspecified)	Variable	Mechanistic	Compartmental	4 age groups	X		X
Ko 2021 (50)	South Korea	Reducing contact	Vaccination (unspecified)	84%	Both	Compartmental	5 age groups	X		X
Lin 2021 (51)	China	N/A	Antivirals	N/A	Mechanistic	Compartmental	4 age groups	X		X
Omae 2021 (52)	Japan	N/A	BNT162b2	Decreased infection by 60 and 92% for 1^st^ and 2^nd^ doses	Mechanistic	Compartmental	N/A	X		X
Yufeng 2022 (53)	China	Reducing contact	N/A	N/A	Mechanistic	Compartmental	N/A	X		X
Zhang 2022 (54)	Pakistan, Bangladesh	Reducing contact	Vaccination (unspecified)	Unspecified	Mechanistic	Compartmental	N/A	X		X
Zhao 2021 (55)	China	N/A	Vaccination (unspecified)	Unspecified	Mechanistic	Compartmental	4 age groups	X		X
Akamatsu 2021 (56)	Japan	Reducing contact	N/A	N/A	Mechanistic	Compartmental	Age as variable		X	X
Alsayed (57)	Malaysia	Reducing contact	N/A	N/A	Mechanistic	Combination	N/A	X		
Chen 2021 (58)	China, Singapore	Border control	N/A	N/A	Mechanistic	Compartmental	N/A	X		
Estadilla 2021 (59)	The Philippines	Contact tracing, reducing contact	Vaccination (unspecified)	Weighted average of available vaccines	Mechanistic	Compartmental	N/A	X		
Hassan 2020 (60)	Bangladesh	Reducing contact	N/A	N/A	Mechanistic	Combination	N/A	X		
Herng 2022 (61)	Malaysia	Reducing contact	N/A	N/A	Both	Combination		X		
Hirata 2022 (62)	Japan	Reducing contact	Vaccination (Pfizer/BioNTech)		Statistical	N/A	N/A	X		
Islam 2021 (63)	Bangladesh	Border control, reducing contact	N/A	N/A	Statistical	N/A	N/A	X		
Kobayashi 2022 (64)	Japan	Reducing contact	Vaccination (unspecified)	Estimated at population level	Mechanistic	Compartmental	Age as variable	X		
Kong 2021 (65)	Global	Border control, reducing contact	N/A	N/A	Mechanistic	Compartmental	N/A	X		
Libotte 2020 (66)	China	N/A	Vaccination (unspecified)	Variable	Mechanistic	Compartmental	N/A	X		
Liu 2022 (67)	China	Contact tracing, border control, reducing contact, hygiene, PPE	N/A	N/A	Both	Compartmental	N/A	X		
Liu 2022 (68)	China	Reducing contact, PPE	Vaccination (unspecified)	Parameterized for susceptibility and infectiousness	Mechanistic	Compartmental	N/A	X		
Lym 2022 (69)	South Korea	Reducing contact	N/A	N/A	Statistical	N/A	2 age groups	X		
Mandal 2020 (70)	India	Border control, reducing contact	N/A	N/A	Mechanistic	Compartmental	N/A	X		
Min 2021 (71)	South Korea	Reducing contact	AstraZeneca, Moderna, Janssen, Pfizer, COVAX facility	52–94%	Mechanistic	Compartmental	3 age groups	X		
Salman 2021 (72)	Malaysia	Reducing contact, education	N/A	N/A	Mechanistic	Combination	N/A	X		
Seok 2022 (73)	South Korea	Contact tracing, reducing contact, Other	Vaccination (unspecified)	48.1–96.1%	Mechanistic	Compartmental	9 age groups	X		
Shen 2022 (74)	China	Reducing contact	Vaccination (unspecified)	Unspecified	Mechanistic	Combination	3 age groups	X		
Wu 2022 (75)	China	Reducing contact	Vaccination (unspecified)	30%	Mechanistic	Statistical	N/A	X		
Xing 2021 (76)	Global	Reducing contact	N/A	N/A	Statistical	N/A	N/A	X		
Yasuda 2022 (77)	Japan	N/A	Vaccination (unspecified)		Mechanistic	Compartmental	2 age groups	X		
Yin 2021 (78)	China	Contact tracing, PPE	N/A	N/A	Mechanistic	Combination	Age as a variable	X		
Yu 2021 (79)	China, Hong Kong	Contact tracing, reducing contact	Vaccination (unspecified)	30, 50, 70%	Mechanistic	Compartmental	N/A	X		
Zhang 2021 (80)	China, Hong Kong	Border control, contact tracing, reducing contact	Vaccination (unspecified)	0–80%	Mechanistic	Combination	4 age groups	X		
Zhao 2021 (81)	China	Education	Vaccination (unspecified)	100%	Mechanistic	Compartmental	N/A	X		
Zhao 2021 (82)	South East Asia	Contact tracing, reducing contact, PPE	Vaccination (unspecified)	70%	Mechanistic	Compartmental	N/A	X		
Zhou 2020 (83)	China	Reducing contact	N/A	N/A	Mechanistic	Compartmental	N/A	X		
Zhu 2021 (84)	Japan	Border control, reducing contact	Vaccination (unspecified)	78.1%	Both	Compartmental	N/A	X		
Zou 2022 (85)	China	Reducing contact	Vaccination (unspecified)	50–90%	Mechanistic	Compartmental	N/A	X		
Hou 2021 (86)	China	Other	N/A	N/A	Mechanistic	Combination	N/A		X	
Jung 2022 (87)	South Korea	Reducing contact, PPE	N/A	N/A	Mechanistic	Compartmental	N/A		X	X
Li 2020 (88)	China	N/A	N/A	N/A	Statistical	N/A	Age as variable		X	
Sunohara 2021 (89)	Japan	Reducing contact	Vaccination (unspecified)	100% against transmission	Mechanistic	Compartmental	3 age groups			X

a Referring to infection unless otherwise specified.

### 3.2. Modeling approaches

Mechanistic models dominated the reports (79.7.0%, 47/59), with fewer statistical (11.9%, 7/59), and combinations of both approaches (8.5%, 5/59) (Table 1). Among the mechanistic models, the majority were based on a compartmental model (61.0%, 36/59). For example, Jung et al. (34) used a susceptible-infected-recovered (SIR) model with time-dependent parameters determined by machine learning. Only one study (1.7%) focused on an agent-based model constructed from statistical data on buildings and the local population to model individual interactions (45); however, a number of studies integrated combination analysis models (18.6%, 11/59), including a study by Shen et al. which compared results from separate SEIR and agent-based models (74).

**Figure 2:**
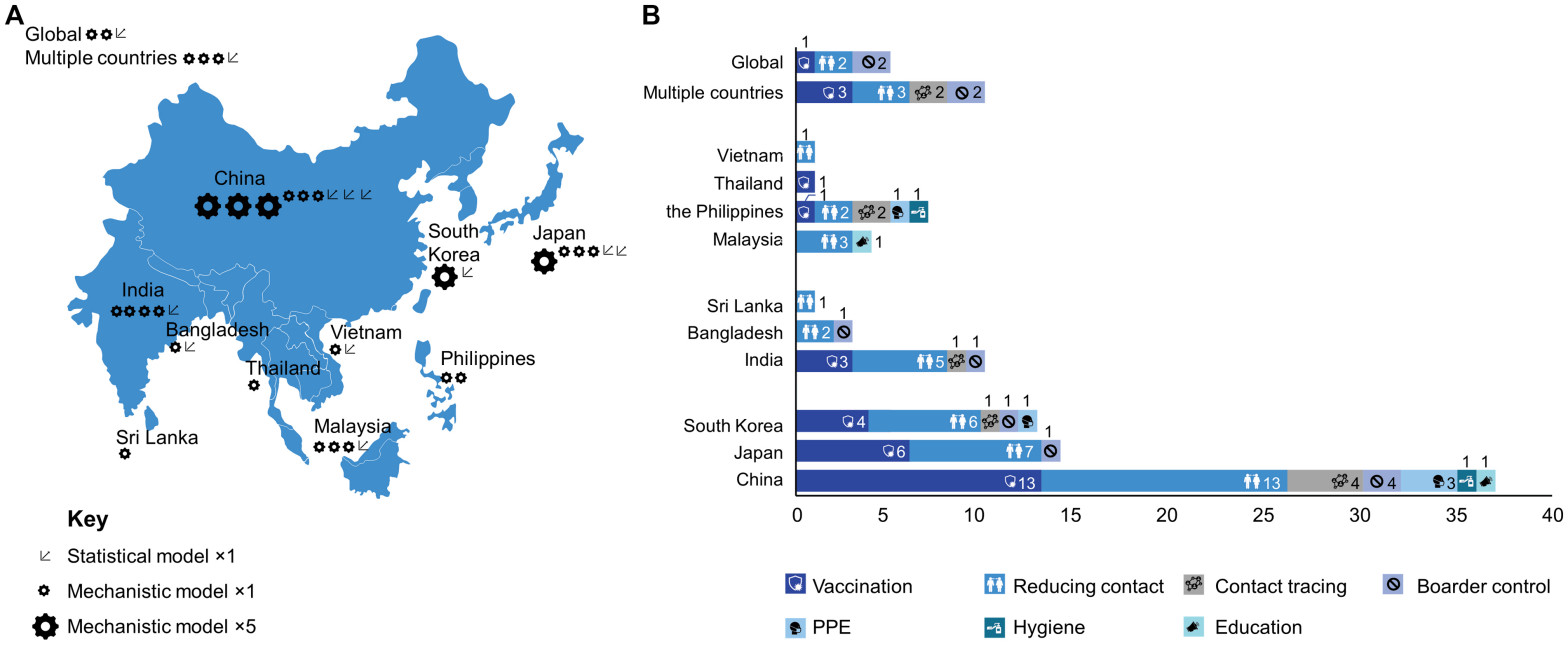
Geographical distribution of reports by **(A)** use of statistical and mechanistic model; **(B)** intervention category.

These proportions were generally similar across the geographic regions (Figure 2A). More than a third of reports accounted for the effects of age in their modeling (37.3%, 22/59), either as an analytical parameter or by separation of specific age groups. For example, Sunohara et al. (89) applied a SEIR model, stratifying the population into three age groups: young (15–49 years), middle (50–64 years), and old (>64 years), ignoring children (0–14 years). A large proportion of studies were prospective (44.1%, 26/59) or included a component of prospective analysis (28.8%, 17/59); completely retrospective studies were less common (27.1%, 16/59).

### 3.3. Interventions

Pharmacological and non-pharmacological interventions were considered in 59.3% (35/59) and 84.7% (50/59) of reports, respectively (not mutually exclusive). Vaccination was the most common pharmacological intervention, considered in 57.6% (34/59) of studies. Notably, vaccination was a factor considered in more than half of the reports from East Asian settings (China, 13/21, 61.9%; Japan, 6/7, 85.7%; South Korea, 4/6, 66.7%), as well as India (3/5, 60.0%) and Thailand (1/1, 100%) (Figure 2B). However, models that incorporated vaccination were either absent or less common than models that incorporated non-pharmacological interventions in many regions of South Asia (Bangladesh, 0/3, 0%; Sri Lanka, 0/1, 0%) and South-East Asia (Malaysia, 0/3, 0%; the Philippines, 1/2, 50.0%; Vietnam; 0/1, 0%). Among studies concerning vaccination, 25.4% (15/59) also considered the influence of age in their models; however, vaccination was explicitly extended to pediatric patients (≥3 years old) in only three studies (32, 38, 55) and adolescents in one study (≥15 years old) (89). In other studies, vaccines were considered to be administered only to the adult population (typically ≥20 years old).

The type of vaccine was generally unspecified or maintained as a ‘hypothetical’ vaccine within the model with effectiveness as a variable. Where vaccine effectiveness was an input parameter in models, a wide range of values for effectiveness in reducing infection were typically used. Several models also parameterized the effectiveness of vaccination for reducing infectiousness and risk of mortality, as well as vaccination type, number of doses, and time from vaccination. Two studies from China specified the use of vaccines based on the inactivated virus, and two studies from Japan specified the use of mRNA vaccines. A study from South Korea compared a variety of different mRNA vaccines and a viral vector vaccine. Traditional Chinese medicine was also considered in one report and antiviral medication in two reports.

A large proportion of the reports that considered non-pharmacological interventions examined the effects of reducing contact (76.3%, 45/59). These kinds of interventions ranged from strict lockdowns on individual movement in regions that report infections (32) to studies that parameterized changes in individual mobility at public transport facilities and spaces (62). Contact tracing (20.3%, 12/59); border control (16.9%, 10/59); and the use of PPE (13.6%, 8/59), i.e., masks and face shields, were also relatively common targets. The effects of hygiene (3.4%, 2/59) and education (3.4%, 2/59) were less commonly measured. For several lower-income regions (i.e., Vietnam, Malaysia, Sri Lanka, Bangladesh), modeling targeted only non-pharmacological interventions (Figure 2B).

### 3.4. Outcomes

#### 3.4.1. Infection

**Figure 3:**
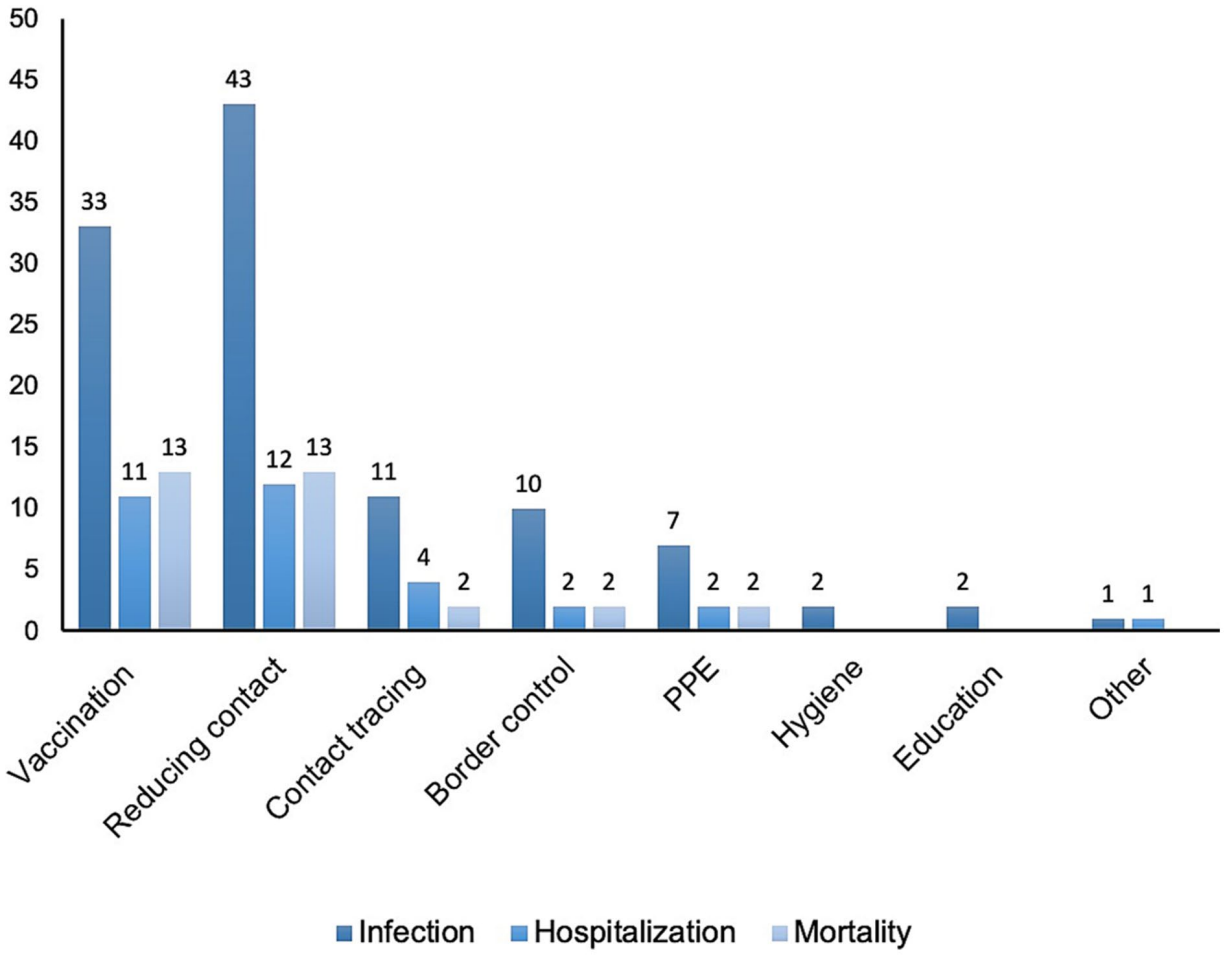
Outcome categories explored in models targeting specific interventions.

Symptomatic infection was a target outcome of almost all models (91.5%, 54/59) (Table 1; Figure 3). Among the 57.6% (34/59) of reports that considered the influence of vaccination, almost all (97.1%, 33/34) examined infection as an outcome. Of the 85.2% (46/54) of reports concerning non-pharmacological impacts on infection (Figure 3), those categorized as reducing contact were most common (93.5%, 43/46), followed by contact tracing (23.9%, 11/46), border control (21.7%, 10/46), and PPE (15.2%, 7/46). Fewer reports considered the effects of hygiene (4.3%, 2/46) or education (4.3%, 2/46) on infection.

Among models that considered infection, 35.2% (19/54) of reports accounted for the influence of age as a factor in their models, including 14 reports of vaccinated populations. With respect to recommendations for prioritizing vaccines to different age groups during the acute stage of the pandemic, only one study in Japan noted potential benefits to infection rates by vaccinating younger people first (89), and two South Korean reports specifically recommended prioritizing older adults first (49, 50). Other reports from China proposed targets for overall population coverage necessary to flatten case numbers. Two studies highlighted the importance of expanding vaccination programs to include young people (32, 55).

#### 3.4.2. Hospitalization and severe disease

Incidences of severe cases of COVID-19 that required medical intervention and/or hospitalization were considered in 30.5% (18/59) of studies (Table 1; Figure 3). Vaccination was considered in 61.1% (11/18) of these studies. A third of reports (33.3%, 6/18) stratified this outcome by age, in which two studies noted that targeted vaccination of older adults or children would decrease hospitalization (32, 38). Additionally, 77.8% (14/18) of reports considered non-pharmacological countermeasures, including reducing contact (66.7%, 12/18), contact tracing (22.2%, 4/18), and border control (11.1%, 2/18).

#### 3.4.3. Mortality

Among the included reports, 30.5% (18/59) considered mortality as a modeling outcome (Table 1; Figure 3); of these, 72.2% (13/18) examined vaccination. Age was a factor in 55.6% (10/18) of reports, and 22.2% (4/18) explicitly noted that vaccination schedules prioritizing older adults during the acute phase of the pandemic would likely reduce overall mortality most effectively (46, 49, 50, 55). One Japanese report suggested that a vaccination strategy that prioritized younger generations might offer benefits in terms of overall mortality (89). However, the stringency of and compliance with non-pharmacological countermeasures was acknowledged to modulate the effectiveness of age-based strategies. Models that incorporated non-pharmacological countermeasures were a focus of 77.8% (14/18) of reports; these countermeasures included contact reduction (72.2%, 13/18), contact tracing (11.1%, 2/18), and border control (11.1%, 2/18).

## 4. Discussion

We examined modeling studies applied to the COVID-19 pandemic in Asia to identify patterns that might guide decision-making on appropriate and timely countermeasures for the ongoing endemic situation and management of future potential SARS-CoV-2 variants of interest. Although, global studies have raised concerns that modeling studies may not give accurate long-term forecasts of numbers of COVID-19 patients, hospitalizations, and deaths, the scenarios generated by such models are still useful for guiding decision-making and resource prioritization (90–92). Across many regions, modeling studies have also played roles in messaging to the public in order to explain and justify otherwise undesirable interventions (93). Our review showed that data-driven modeling studies play a critical role in the understanding of disease and various preventive policies in Asia.

We identified more reports from East Asian nations, particularly dominated by reports from China. However, the strong tendency to use mechanistic modeling was generally reflected across all regions. Despite the considerable differences in public health infrastructure and substantial variations in population densities, income levels, and sociocultural aspects (82), the common use of mechanistic cohort models across regions suggests that this approach is universally attractive for understanding general disease dynamics and the effectiveness of different interventions (94). This choice may also reflect the intuitive nature of this type of model and the ability to easily develop tools suitable for use by policymakers. Other key advantages of mechanistic models include the insights into the disease that might be gained, particularly from validation of the modeling against real-world evidence. Hence, mechanistic disease-spread models are often used as a basis for cost-effectiveness analyses (95). Additionally, the broad range of symptoms that manifest in COVID-19 introduces considerable uncertainty into national reporting of disease statistics; thus, more sophisticated models (i.e., agent-based models) may not offer particular benefits in accuracy based on available input data.

Particularly early in the pandemic, prior to the wider availability of vaccines, models examined the influence of non-pharmacological interventions on disease-related outcomes. Models set in South and South-East Asian regions were particularly focused on non-pharmacological interventions rather than vaccination, which may reflect the timing of this literature search and the slower vaccine approval, acquisition, and administration in these regions. The majority of models examined here looked at reducing contact through restrictions of movement and gatherings. This focus may reflect the economic disruption caused by these interventions. Although such measures are acknowledged to be effective in reducing disease transmission, their impact is difficult to assess in terms of financial burden and sustainability and compliance; thus, it remains challenging to gauge the accuracy of modeling applied to these measures (96). Few studies here included explicit cost-effectiveness assessments for managing COVID-19 with non-pharmacological interventions or vaccination strategies (47, 88, 89). Whereas vaccination is considered to be cost-effective in terms of treatment costs, strictly enforced countermeasures can pose considerable socio-economic and mental health burdens on populations, which are not fully considered in any of the models identified here (97, 98).

Following the introduction of vaccines from late 2020, reports more often incorporated the effects of vaccination into models. Although information on the specific vaccines used was often limited, actual administration patterns may be inferred from local approval schedules and government procurement policies. Whereas China has almost exclusively used inactivated vaccines (e.g., CoronaVac, Sinovac Biotech), Japan and South Korea granted early approval for mRNA vaccines (e.g., Comirnaty, Pfizer–BioNTech, Pfizer Inc.; Spikevax, Moderna Inc), which have been widely administered. In India, a viral vector vaccine (Vaxzevria, Oxford-AstraZeneca) was used extensively; and across other South and South-East Asian countries, a variety of vaccine types have been deployed due to varying availability and access.

Early recommendations on vaccination strategies were broadly similar, regardless of the vaccination type and local situation, emphasizing the need for rapid distribution and high overall population coverage. Models examining vaccination identified here underline the importance of rapidly achieving 67–83% population coverage with effective vaccines to reduce infection, hospitalization, and/or mortality rates (99). Direct comparisons of modeling results with actual real-world data are complicated by various factors. The methods of diagnosing infection have developed from polymerase chain reaction (PCR) testing initially, through to antigen rapid testing later in the pandemic. Additionally, the implementation of home-based recovery programs in some regions may have underestimated the actual number of infections and hospital admissions (100). Nevertheless, predictions of vaccination outcomes from Asian models have been widely confirmed by lower rates of infection and hospitalization among vaccinated individuals and higher excess mortality among unvaccinated adults across these regions (101, 102).

Reports diverged in terms of specific recommendations for prioritizing vaccination of different age groups. In China and South Korea, where strict social measures were implemented alongside widespread use of inactivated virus or mRNA vaccines, respectively, the majority of models recommended priority vaccination of older adults, a groups that has being prioritized for vaccination since the beginning of the pandemic (103). This is consistent with results from early global modeling studies that also prioritized older adults (90, 104, 105).

In Japan, which implemented voluntary social distancing and predominantly administered mRNA vaccines, one study noted a potential for benefits in terms of indirectly reducing mortality by targeting younger age groups to reduce overall transmission, depending on the strength of the lockdown (89). The actual vaccination strategy implemented in Japan, however, prioritized older adults, which may have helped reduce the hospital burden but could have been suboptimal in reducing transmission, particularly in the context of weakly enforced social measures. However, attitudes towards COVID-19 suggested remarkable concern among the Japanese public, which contributed to good compliance with government advice to wear face masks, social distance, and accept vaccination/boosters (106). Globally, the attitudes of populations towards the disease, trust in policy makers, and enforcement of interventions are factors that may need to be accounted for in modeling studies (107).

The evolution of new viral strains may also influence vaccination strategies. The main variants of SARS-CoV-2 circulating in different regions have changed, from the original strain prevalent in 2020, to four main variants of concern – the Alpha, Beta, and Delta strains, common across Asia in 2021 – through to the Omicron variants from 2022 (3, 4, 108). Notably, the changes from the original variant to the Delta variant and then later to the Omicron variant were accompanied by increases in R_0_ values, from 2.79 to 5.08 to 8.20, respectively (109, 110). The recently emerged Omicron subvariants, such as XBB and XBF, appear to be key drivers of infection waves, although they appear to pose lower risks of mortality (111). The emergence of new variants presents challenges for modeling in terms of shifting profiles for infectiousness, severe disease and mortality, and breakthrough infection against different vaccines and vaccination statuses (i.e., number/type of vaccination and time since last booster) (112). For example, individuals with hybrid immunity (developed through both infection and vaccination) might experience greater and more sustained protection, which might allow for longer intervals between boosters (113).

The outcomes of many models have anticipated that sufficiently high vaccination coverage in a population might ‘break’ transmission, allowing for the relaxation of non-pharmacological interventions (71, 114). In reality, vaccinated populations in Asia have continued to fall short of the high proportions predicted to be necessary to confer ‘herd immunity’ despite large numbers having received initial vaccinations and boosters (115). The waning effectiveness of mRNA vaccination/booster shots over time (after approximately 20 weeks) – particularly among older adults, who typically have weakened immune systems – may increase rates of infection (116). The potential impact of immune imprinting also motivates further studies to understand the effects and limitations of boosters (117). Reduced effectiveness of vaccines against Omicron strains might also shift initial targets for vaccination coverage in a population upwards, obstructing the relaxation of non-pharmacological interventions (118). Ongoing viral transmission from viral shedding from convalescent individuals, in addition to cessation of mask usage, may contribute to future surges of the disease.

There is also a need to deeply understand the importance of using vaccines that confer broad protection in the Omicron era, such as bivalent vaccines (119). Recent real-world evidence on bivalent vaccines points to additional protection, primarily against hospitalization, among older adults with monovalent vaccines; however, there may also be some additional benefits against symptomatic disease in other age groups during Omicron subvariant circulation (120). The high-priority populations are defined as older adults, adults with significant comorbidities, children or adults with immunocompromising conditions, pregnant women, and frontline health workers.

We noted that few studies (32, 38, 55) examined here modeled the vaccination of pediatric populations in Asia. Modeling of the impact of vaccinating children (5–11 years) in Europe has been proposed as a route to the relaxation of restrictions in schools (121). Modeling studies have also suggested that extending vaccination to children may reduce hospitalization and mortality across all age groups in developing countries (122). The latest update from the WHO’s Strategic Advisory Group of Experts on Immunization (SAGE) on COVID-19 vaccination continues to prioritize the populations at greatest risk of mortality or severe disease to safeguard healthcare systems. As a low priority group, children are not routinely recommended for vaccination and boosters, but are instead determined by individual countries’ evaluation of the burden, cost, and effectiveness (123). Although data on the impact of COVID-19 among children are lacking overall, there appears to be greater risk of mortality and hospitalization in low and middle-income countries (124). Thus, there is a need for further studies focused on the local burden of COVID-19 disease among younger populations in Asia, where future modeling studies might also guide decision-making on the impact of vaccinations (121, 122, 125).

Moving into a period where more vaccines are approved and made accessible, the hesitancy of uptake is likely to pose an ongoing barrier to booster strategies and sustainable protection (126). Among children and their caregivers, common reasons for fear and hesitancy to receive a vaccine include assumptions that the vaccine has side effects and may not be safe (18). Adults may also be complacent that the COVID-19 situation is no longer severe, and/or a negative perception of the vaccine, government, and/or pharmaceutical company may discourage them from receiving initial vaccination and/or boosters (17, 18). General fatigue over the topics of COVID-19 and vaccines and a wider circulation of misinformation on vaccines may contribute to these attitudes (127). Some reports examined here accounted for vaccine hesitancy, but this factor will likely be more important in future COVID-19 models, where the majority of a population have been vaccinated but coverage falls short of requirements for herd immunity. Modeling may also need to account for the impacts of educational interventions to address gaps in knowledge and attitudes among hesitant groups. Therefore, from a public health perspective, it is crucial to understand the drivers of vaccine uptake and vaccine hesitancy; this might help to identify groups that might have lower than average uptake and plan accordingly. Such pockets of immunity gaps and high susceptibility in the population could result in small-scale outbreaks that reduce the effect of population immunity. We predict that relaxation of control measures might be associated with new waves of infection and associated deaths; however, these outcomes will be reduced by increased levels of vaccine-derived immunity as well as hybrid immunity from infections in vaccinated individuals in the population.

The evolving nature of the COVID-19 situation especially motivates a dynamic vaccine-development pipeline to deliver more effective options against new strains of SARS-CoV-2 (128, 129). Furthermore, in an endemic scenario, non-vaccine pharmacological interventions are likely to become important for the treatment of severe disease. Here, two studies were identified that considered the impact of antiviral treatments, which may reflect the regional timeline for approval of such medicines. The introduction of monoclonal antibodies may help to manage cases of severe COVID-19 and improve these outcomes (10).

There are several limitations to this study. First, the scope of the literature search, particularly the focus on journal articles published in the English language, may have omitted more recent pre-print and local-language documents. The specific search terms may also have introduced bias into the types of modeling studies captured here. Furthermore, the large diversity of regional situations, model designs, interventions, and outcome targets limit the ability to systematically extract relevant quantitative data from these reports. Additionally, few studies examined the influence of pharmacological interventions other than vaccines. As common limitations of all modeling studies, there is considerable uncertainty in many parameters such as population immunity, infectious rate, and contact and the level of health-related behaviors in study populations (130). Thus, modeling studies are subject to unreliable predictions on the impact on outcomes of interest.

## 5. Conclusion

Computational modeling is an important tool to address a limited understanding of SARS-CoV-2 epidemiology, and the quantitative estimates of the duration of protection from infection and vaccinations. Whereas non-pharmacological interventions have had an early role in managing health outcomes of the COVID-19 pandemic, modeling studies underline the importance of vaccines. High population coverage and vaccine effectiveness are key to mitigating the outcomes of the disease and supporting the relaxation of disruptive restrictions on movement. Compartmental mechanistic modeling offers an adequate approach to projecting disease outcomes across large populations; however, future models may need to account for complications from evolving variants and vaccination statuses/inclinations within populations, age group stratification, especially including pediatric populations. Deeper consideration of the socioeconomic burdens associated with strict non-pharmacological interventions may also be useful for policymakers and further underscore the cost-effectiveness and social benefits of vaccination programs. In the near future, increasing vaccine coverage, particularly among at-risk populations and through outreach to vaccine-adverse groups, may help to ease infection and severe disease. The rapidly evolving nature of the virus also motivates the development of new vaccines and alternative therapies. Future administration strategies may also be nuanced by competing needs to reduce overall transmissibility or severe disease burden in high-risk groups.

## Author contributions

KT, JS, EO, JY, and MK: conceptualization and methodology. KT, JS, JY, MK, and CM: formal analysis and data curation. KT, JS, JY, MK, EO, CM, and HO: validation, writing, reviewing, and editing of manuscript. All authors contributed to the article and approved the submitted version.
