# Supplementary material for: Impact of vaccination and non-pharmacological interventions on COVID-19: a review of simulation modeling studies in Asia
Source: Front Public Health. 2023 Sep 25;11:1252719. doi: 10.3389/fpubh.2023.1252719 (PMC10560858; doi:10.3389/fpubh.2023.1252719)
Supplement: Supplementary file 1 [file Table_1.docx]

**Supplementary Table 1: Search terms**

((Susceptible-Infected-Recovered) OR (SIR) OR (Susceptible-Exposed-Infected-Recovered) OR (SEIR) OR (Susceptible-Infected-Recovered-Death) OR (SIRD) OR (Susceptible-Exposed-Infected-Recovered-Death) OR (SEIRD) OR (simulation) OR (agent-based) OR (silico) OR (mechanic*) OR (stochastic) OR (state transition) OR (discrete event simulation) OR (DES) OR (deterministic) OR (renewal equation) OR (branching) OR (decision analytic) OR (Markov cohort) AND (model)) AND ((prevalence) OR (epidemiology) OR (incidence) OR (hospitalization) OR (infection) OR (death) OR (mortality)) AND ((policy) OR (vaccination) OR (immunization) OR (program*) OR (intervention)) AND ((Asia) NOT (Middle-East) NOT (Europe) NOT (Africa) NOT (America)) AND (COVID-19) NOT (Clinical Trial[Publication Type] OR (Randomized Controlled Trial[Publication Type])
